# Identification of jasmonic acid-associated microRNAs and characterization of the regulatory roles of the miR319/TCP4 module under root-knot nematode stress in tomato

**DOI:** 10.1093/jxb/erv238

**Published:** 2015-05-22

**Authors:** Wenchao Zhao, Zilong Li, Jingwei Fan, Canli Hu, Rui Yang, Xin Qi, Hua Chen, Fukuan Zhao, Shaohui Wang

**Affiliations:** ^1^Beijing Key Laboratory for Agricultural Application and New Technique, Plant Science and Technology College, Beijing University of Agriculture, Beijing, 102206, China; ^2^Biological Science and Engineering College, Beijing University of Agriculture, Beijing, 102206, China

**Keywords:** Deep sequencing, jasmonic acid, miRNAs, miR319/TCP4, root-knot nematode, tomato.

## Abstract

MicroRNAs (miRNAs) are important transcriptional and post-transcriptional modulators of gene expression that play crucial roles in the responses to diverse stresses. To explore jasmonic acid (JA)-dependent miRNA-mediated regulatory networks that are responsive to root-knot nematode (RKN), two small RNA libraries were constructed from wild-type (WT) and JA mutant (*spr2*) plants. A total of 263 known miRNAs and 441 novel miRNAs were significantly regulated under RKN stress in the two libraries. The spatio-temporal expression of candidate miRNAs and their corresponding targets were analysed by qRT-PCR under RKN stress. A clear negative correlation was observed between miR319 and its target TEOSINTE BRANCHED1/CYCLOIDEA/PRO-LIFERATING CELL FACTOR 4 (TCP4) in leaf, stem, and root under RKN stress, implying that the miR319/TCP4 module is involved in the systemic defensive response. Reverse genetics demonstrated that the miR319/TCP4 module affected JA synthetic genes and the endogenous JA level in leaves, thereby mediating RKN resistance. These results suggested that the action of miR319 in serving as a systemic signal responder and regulator that modulated the RKN systemic defensive response was mediated via JA. The potential cross-talk between miR319/TCP4 and miR396/GRF (GROWTH RESPONDING FACTOR) in roots under RKN invasion is discussed, and a predictive model regarding miR319/TCP4-mediated RKN resistance is proposed.

## Highlight

Screening revealed that the action of miR319/TCP4 in serving as a systemic defensive responder and regulator that modulated the RKN systemic defensive response was mediated via JA.

## Introduction

Small RNAs (sRNAs) are a class of short (20–30 nucleotide), endogenous, non-coding RNAs that play important roles in plant growth, development, and adaptation to environmental stress (Lu *et al.*, 2008; Ruth *et al.*, 2010). sRNAs primarily include microRNAs (miRNAs) and small interfering RNAs (siRNAs). In plants, miRNAs have been shown to regulate plant growth and development under various biotic and abiotic stresses, such as drought stress in *Oryza sativa* and *Populus euphratica* (Lu *et al.*, 2008; Zhou *et al.*, 2010; Li *et al.*, 2011), heat stress in *Brassica rapa* (Yu *et al.*, 2012), and heavy metal stress from copper (Waters *et al.*, 2012), aluminium (Chen *et al.*, 2012), and cadmium (Ding *et al.*, 2011). miRNAs were also reported to be involved in plant–parasite interactions. In plants, miR393 was first reported to play a role in plant antibacterial PTI (pattern-triggered immunity) by regulating the auxin signalling pathway (Navarro *et al.*, 2006). High-throughput sequencing identified a range of miRNAs, such as miR156, miR159, miR172, miR319, miR393, and miR396, in response to *Pseudomonas syringae* (Zhang *et al.*, 2011). Recently, a study of tomato infection by *Cucumber mosaic virus* (CMV) revealed 79 miRNAs and 40 predicted candidate miRNAs that were responsive to CMV infection, including miR156, miR159, miR172, miR319, mi393, and miR396 (Feng *et al.*, 2014). Shen *et al.* (2014) revealed that 62 miRNAs are responsive to *Verticillium longisporum* infection, including the conserved miR319 family. This growing body of evidence strongly suggests that miRNAs play roles in plant defences against viruses (Kasschau *et al.*, 2003; Bazzini *et al.*, 2007; Navarro *et al.*, 2008), fungi (Lu *et al.*, 2007), and bacteria (Jagadeeswaran *et al.*, 2009; Xin *et al.*, 2010; Zhang *et al.*, 2011). In addition, a negative correlation between miRNA abundance and their targets was observed in *Arabidopsis* roots after infection with cyst nematode, leading to the down-regulation of miR156, miR159, miR172, and miR396 (Hewezi *et al.*, 2008). Further studies have shown that the miR396/GROWTH RESPONDING FACTOR 1 (GRF1)/GRF3 regulatory module acts as a developmental regulator in the formation of syncytia during cyst nematode infection (Hewezi *et al.*, 2012). Since cyst nematodes and root-knot nematodes (RKNs) feed on syncytia and giant cells, respectively, the potential mechanisms of formation of feeding sites are distinct (De Almeida Engler *et al.*, 2011; Kyndt *et al.*, 2013). To date, however, studies of the link between RKN invasion and miRNA-modulated defence responses are extremely limited.

Jasmonic acid (JA) as a systemic signalling molecule is effective against tomato RKN disease and can reduce the number of root knots from nematode invasion (Cooper *et al.*, 2005; Fujimoto *et al.*, 2011; Nahar *et al.*, 2011; Fan *et al.*, 2014). JA is synthesized via the octadecanoid pathway in leaves, which involves the translocation of lipid intermediates from chloroplast membranes to the cytoplasm and later into peroxisomes (León, 2013). However, JA-mediated RKN resistance occurs in roots. Therefore, synthetic JA and methyl jasmonate (MeJA), which are considered to be long-distance signalling compounds, can be transported from shoots to roots via the vasculature to activate a series of defence responses against RKN invasion (Heil and Ton, 2008). JA has been shown to be transported in the phloem in response to pathogen infection (Schilmiller and Howe, 2005; Lough and Lucas, 2006; Thorpe *et al.*, 2007; Truman *et al.*, 2007). Therefore, the phloem is considered to be a hub that connects shoots and roots and in which miRNAs potentially play crucial roles.

Recent studies indicate that miRNAs are responsive to JA. For example, exogenous MeJA down-regulates miR156, miR168, miR169, miR172, miR172, miR396, miR480, and miR1310 and up-regulates miR164 and miR390 in Chinese yew (Qiu *et al.*, 2009). In one study, 49 known miRNAs, 15 novel miRNAs, and three tasiRNA (*trans*-acting small interfering RNA) families were induced in JA-treated wild-type (WT) *Arabidopsis*, whereas one new miRNA, one tasiRNA family, and 22 known miRNAs were repressed in the JA-deficient *aos* mutant (Zhang *et al.*, 2012). Additionally, in *Nicotiana attenuata*, insect herbivores have been shown to alter sRNA transcriptomes related to phytohormone (JA and JA-Ile) signalling (Pandey *et al.*, 2008), which implies that sRNAs probably serve as regulators in JA-mediated biotic stress responses.

The present work was planned to identify RKN-responsive JA-mediated miRNAs and interpret the roles of candidate miRNA. To the authors’ knowledge, it is the first exploration of the responsive miRNAs under RKN stress. Sequencing of sRNAs was performed on phloem tissue after RKN inoculation in WT and *spr2* (suppressor of prosystemin-mediated response 2, JA-deficient) tomatoes. A total of 263 known and 441 novel differentially expressed miRNAs were identified, and the roles of the JA-mediated miR319/TEOSINTE BRANCHED1/CYCLOIDEA / PRO-LIFERATING CELL FACTOR 4 (TCP4) module in the RKN-defensive response were demonstrated. Previous evidence has demonstrated that miR319s and their targets participate in abiotic stress responses and play positive roles in high salinity and drought stress resistances (Sunkar and Zhu, 2004; Liu *et al.*, 2008; Lv *et al.*, 2010; Zhou *et al.*, 2010; Thiebaut *et al.*, 2012). The outcomes of this study would provide novel insight into the functions of the miR319/TCP module in plants.

## Materials and methods

### Plant materials and biotic stress treatment

Tomato cultivar CM (*Solanum lycopersicum* var Castlemart, WT) and its mutant *spr2* (deficient in JA biosynthesis because *Spr2* encodes a chloroplast fatty acid desaturase involved in JA biosynthesis; Li *et al.*, 2003) were grown in a greenhouse with a day/night temperature of 25/18 °C, an air relative humidity (RH) of 60/70%, and a photosynthetic photon flux density (PPFD) of 500 μmol^–2^ s^–1^ for 10h d^–1^. RKN isolates were clonal populations established from a single female. They were maintained on tomato UC82 plants. When the seedlings had a total of four spread true leaves, nematodes were inoculated at 5000 per plant near the root. Leaves, phloem (stem), and roots were harvested from the WT and *spr2* at 6, 12, 24, and 72h after inoculation. For hormone treatment, leaves were collected from the WT after JA treatment at 0h and 6h. These samples were used for quantitative reverse transcription–PCR (qRT–PCR) analysis of miRNAs and their targets. The infected seedlings were harvested after 20 d for high-throughput sequencing, and the primary stems were cut 10cm above the bottom of the root. The phloem tissue was quickly peeled off with forceps and a blade treated with DEPC (diethylpyrocarbonate), and the phloem tissue was placed in RNA-free tubes, flash-frozen in liquid nitrogen, and stored at –80 °C.

To investigate the role of *miR319* in response to RKN (*Meloidogyne incognita*) stress, PCR was performed to confirm that the genomes of the plants under study did not contain *Mi-1* (Supplementary Fig. S1 availbale at *JXB* online), which confers effective resistance against RKNs in tomato (*S. lycopersicum* L.) (Seah *et al.*, 2004). The transgenic tomatoes overexpressing *Arabidopsis* pre-miR319a or tomato *TCP4* (*LA*) were in the M82 background. To overexpress miR319 or *LA*, the *op:gene* plants (*opmiR319* or *opLA* ^*m*^) were crossed with *FIL:LhG4* (*pFIL*), and hybrid F_1_ seedlings were used for further study. The relative expression levels of JA biosynthetic genes in the WT and transgenic tomatoes (miR319-oe, LA^m^-oe) were analysed at 0, 6, 12, and 24h after inoculation using qRT–PCR.

### Small RNA library construction and high-throughput sequencing

Total RNA was extracted with the TRIzol reagent according to the manufacturer’s instructions. RNA quality was assessed with an Agilent Technologies 2100 Bioanalyzer. The RIN (RNA integrity number) was 7.2 for the WT and 6.2 for the *spr2* mutant. Total RNA from WT and *spr2* plants was prepared for sRNA sequencing-by-synthesis according to the protocols and standards for Illumina preparation at the Beijing Genomics Institute (BGI; Shenzhen, Guangdong, China). Briefly, RNA fragments of 18–30 nucleotides were isolated from the total RNA by electrophoretic separation via 15% TBE–urea denaturing PAGE. The 5ʹ RNA adaptor (5ʹ-GUUCAGAGUUCUACAGUCCGACGAUC-3ʹ) and 3ʹ RNA adaptor (5ʹ-pUCGUAUGCCGUCUUCUGCUUGUidT-3ʹ; p, phosphate; and idT, inverted deoxythymidine) were ligated to the isolated sRNAs using T4 RNA ligase (TaKaRa). The products were separated and purified at each step by TBE–urea PAGE. The 62–75 nucleotide ligated products were subsequently transcribed into cDNA and amplified by PCR, followed by purification and separation. The purified DNA fragments were used for sequencing with an Illumina 1G Genome Analyzer, an sRNA digital analysis system.

### Small RNA annotation

Raw sequence reads were processed into clean full-length reads by discarding low-quality reads and corrupted adaptor sequences [reads <18 nucleotides, reads >30 nucleotides, reads with a poly(A), 3ʹ adaptor, or 5ʹ adaptor contaminants, and reads without a ligated adaptor]. Adaptors were then trimmed from the remaining high-quality sequences. The distribution of small RNA lengths was analysed, and the two samples were compared to identify common and genotype-specific transcripts. The Short Oligonucleotide Analysis Package (SOAP; http://soap.genomics.org.cn) was used to annotate the sRNA sequences, which were then mapped to tomato TIGR reference sequences. After comparing the unique sequences with the Rfam 9.1 (http://ftp.selab.janelia.org/pub/Rfam) and NCBI GenBank databases (http://ftp.ncbi.nlm.nih.gov), all rRNAs, scRNAs, snoRNAs, snRNAs, and tRNAs were discarded. The NCBI GenBank annotation has priority over Rfam (9.1). The sRNAs were then classified and annotated. The total rRNA content served as a quality standard.

### Identification of known and novel differentially expressed miRNAs

The remaining sequences were analysed by BLAST searches of miRBase16.0 (http://www.mirbase.org/index.shtml) for matches to precursors or mature miRNAs; these known miRNAs came from all the plants. To evaluate the expression patterns of known miRNAs, a statistical analysis was used to determine the significance of known differences in miRNA expression between the WT and *spr2* strains. Log_2_ ratios and scatter plots were used to compare miRNA expression, and fold change=log_2_ (treatment/control). Novel miRNAs were predicted according to the precursor hairpin secondary structure in Mireap (https://sourceforge.net/projects/mireap/). Briefly, potential novel miRNAs in the tomato genome were predicted using the following criteria: (i) the sequences of miRNA precursors folded into stem–loop structures that contain an ~21 nucleotide mature miRNA sequence on one arm and the miRNA* derived from the opposite arm to form a duplex with two nucleotides for the 3ʹ overhang; (ii) the Dicer PAZ structural domain, which binds the end of double-stranded RNA, recognizes the two-nucleotide overhang of the stem–loop structure, and cuts it into miRNAs; and (ii) the miRNAs have lower folding free energies than random sequences with the same nucleotide content, so the stem–loop structures have a folding free energy of at most –18 kcal mol^–1^ (the lowest free energy is Mfe ≤ –18 kcal mol^–1^). χ^2^ tests were used to determine the statistical significance of the differences between the two libraries. Compared with the WT group, the known and novel differentially expressed miRNAs with fold changes >2.0 and a *P*<0.01 were selected.

### Identification and KEGG enrichment analysis of miRNA target genes

The targets of known and novel miRNAs were predicted using psRNATarget (Dai and Zhao, 2011). All the predictive targets were assigned KEGG terms (Moriya *et al.*, 2007), and a search was carried out for significantly enriched KEGG terms compared with the entire transcriptome background.

### Real-time PCR assay

A time-dependent expression analysis of selected miRNAs and their targets was performed by stem–loop RT–PCR and qRT–PCR. For stem–loop RT–PCR, total RNA was reverse-transcribed into cDNA using the Super-Script first-strand synthesis system (Invitrogen) according to the manufacturer’s instructions. The cDNA was used as a template to perform real-time PCR with gene-specific primers and SYBR Green Mix (TaKaRa). Target expression levels were normalized by using tomato *U6* as an internal reference. Stem–loop reverse transcription was performed according to a previous protocol (Varkonyi-Gasic *et al.*, 2007). For qRT–PCR, reverse transcription was performed using TransScript First-Strand cDNA Synthesis SuperMix (TransGen) following the manufacturer’s instructions. The resulting cDNA was analysed by relative quantitative PCR in the presence of SYBR Premix Ex Taq II (TaKaRa) in a Bio-Rad iCycler, with β-actin as the internal control. After the PCR, a melting curve was generated by gradually increasing the temperature to 95 °C to test the amplicon specificity. The primers for stem–loop RT–PCR and qRT–PCR are listed in Supplementary Table S1 at *JXB* online. All reactions were performed in triplicate.

### Acid fuchsin staining

The root systems of each plant were harvested at 35 d after RKN infection and then submerged in a 15% solution of McCormick’s red food colour (Thies *et al.*, 2002) for 15–20min to stain the egg masses. The root systems were carefully rinsed under running tap water and evaluated for gall severity and egg mass production.

### JA level determination

Plant tissue was homogenized in liquid nitrogen, and ~100mg of fresh leaves was sealed in 2ml tubes (if >100mg, in 5ml tubes). A 0.5ml aliquot of extraction buffer (2:1:0.005, isopropanol:water:concentrated HCl) was added to each sample with d5-JA (40ng CDN Isotopes) as internal standards. The samples were homogenized for 45 s, followed by agitation for 30min at 4 °C. CH_2_Cl_2_ (1ml) was added to the samples, followed by agitation for another 30min (the CH_2_Cl_2_ extraction step was performed on a vortexer with a 30 sample attachment at room temperature) and then centrifuged at 13 000*g* for 5min. After centrifugation, two phases formed, and the plant debris was in the middle of the two layers. The aqueous phase was discarded, and the lower layer was collected, concentrated in a drying machine, and re-solubilized in MeOH+water. If the solution contained precipitates, it was transferred to a glass tube and centrifuged at 13 000 *g* for 30min. Afterwards, 5–10 μl was injected into the column for analysis (Pan and Wang, 2009).

## Results

### An overview of sRNA sequencing

To identify the miRNAs involved in JA-mediated RKN resistance in tomato, two sRNA libraries from WT and *spr2* mutant tomatoes were constructed. Solexa sequencing technology was used to investigate the expression abundance of sRNA in the two libraries, which generated a total of 17 753 883 and 11 251 489 reads in the WT and *spr2*, respectively. After removing the adaptors and low-quality tags, 16 694 025 (representing 6 195 892 unique reads) and 10 439 048 (representing 2 520 926 unique reads) clean reads remained for the WT and *spr2* libraries, respectively. In total, 5 019 256 (WT) and 1 921 123 (*spr2*) reads mapped perfectly to the *S. lycopersicum* genome. Thereafter, the non-coding RNAs, including rRNAs, tRNAs, snRNAs, and snoRNAs, were annotated and removed. Querying the remaining sequences against miRBase 16.0 identified 38 738 (WT) and 17 546 (*spr2*) unique reads that matched known miRNAs (Table 1).

**Table 1.** *A statistical analysis of sequencing reads from WT and* spr2 *sRNA libraries*

Library type	WT	*spr2*
Total raw reads	17 753 883	11 251 489
Total clean reads	16 694 025	10 439 048
Unique clean reads	6 195 892	2 520 926
Total clean reads mapped to genome	14 700 657	8 944 510
Unique clean reads mapped to genome	5 019 256	1 921 123
Total miRNA reads	38 738	17 546

**Fig. 1.**
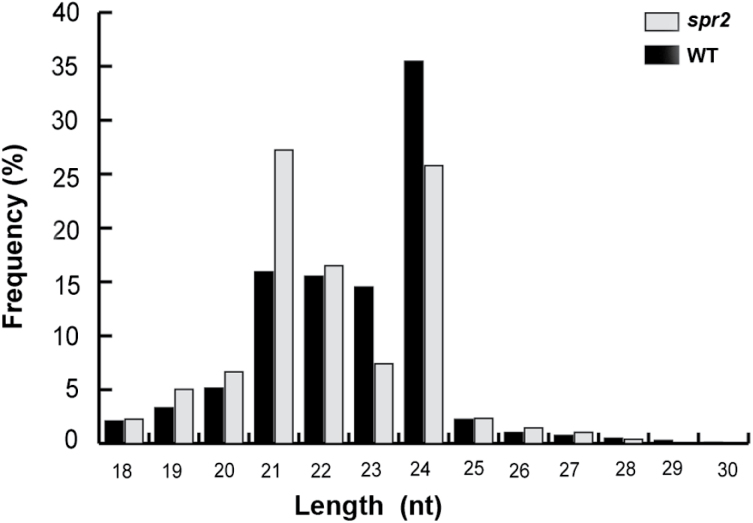
The length distribution and abundance of sRNAs in the libraries of WT (blank bars) and *spr2* (grey bars).

The 21 and 24 nucleotide RNAs were the most abundant sRNA species (Fig. 1). The sRNAs ranged from 21 to 24 nucleotides in length and comprised 81.46% of the WT and 76.94% of the *spr2* transcripts, which was consistent with evidence found in the conifers *Pinus contorta* and *Taxus chinensis*, for which >50% of the sequenced sRNAs were 21 or 24 nucleotides (Qiu *et al.*, 2009). In contrast to the results found in *T. chinensis* (Qiu *et al.*, 2009), the percentage of 24 nucleotide sRNAs was much higher than the percentage of 21 nucleotide sRNAs in the WT in this study. The sRNAs had a strong bias for 21 nucleotide lengths in the *spr2* strain, almost twice the rate in the WT strain. In addition, 24 nucleotide sRNAs dominated the WT strain, which was consistent with previous deep sequencing studies in which Hi-JA *Arabidopsis* exhibited a bias for 24 nucleotide sRNA populations (Zhang *et al.*, 2012).

### Identification of differentially expressed known and novel miRNAs

**Fig. 2.**
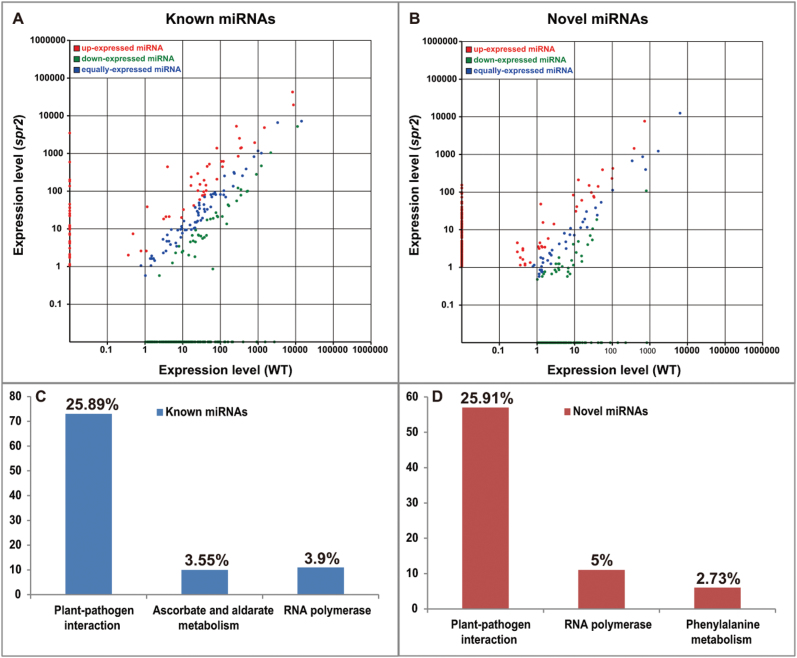
A scatter plot of differentially expressed known (A) and novel (B) miRNAs. The abscissa represents the expression level of miRNAs in the WT; the ordinate represents the expression level of miRNAs in *spr2*. Dots appearing in the shaded sections represent differential expression miRNAs. Top left, increased expression; bottom right: reduced expression. (C, D) The number of known and novel differentially expressed miRNAs in KEGG pathways. The corresponding percentage is shown above the column. (This figure is available in colour at *JXB* online.)

To identify the differential expression of known miRNAs from the two libraries for the WT and *spr2* strain, clean reads were used to compare known plant miRNA precursors or mature miRNA sequences using miRBase 16.0. Novel miRNAs were predicted according to the precursors’ hairpin secondary structure in Mireap and by using the miRNA/miRNA* criteria. The miRNA expression was considered to be significantly up-regulated or down-regulated with fold changes >2.0 or fold changes < –2.0, respectively, with *P*≤0.001. In total, 263 known and 441 novel miRNAs were differentially expressed (Supplementary Tables S2, S3 at *JXB* online). Scatter plots were used to compare the expression of known and novel miRNAs (Fig. 2A, B).

### Target prediction and KEGG enrichment of differentially expressed miRNAs

miRNAs regulate the expression of specific genes via hybridization to mRNA transcripts to promote RNA degradation, inhibit translation, or both (Krol *et al.*, 2010). Known and novel miRNA sequences were searched against tomato genomic sequences on the psRNATarget webserver. Among the 263 known miRNAs, 131 could be searched for their potential targets, yielding 473 genes (Supplementary Table S4 at *JXB* online). Of the 441 novel miRNAs, 161 miRNAs had 359 predicted targets (Supplementary Table S5). KEGG pathway analysis indicated that the significantly enriched pathways were ‘plant–pathogen interaction’ (ko04626) and ‘ascorbate and aldarate metabolism’ (ko00053) for known miRNAs and ‘plant–pathogen interaction’ (ko04626) and ‘RNA polymerase’ (ko03020) for novel miRNAs (Fig. 2C, D; Supplementary Table S6). Therefore, the focus of further study was on miRNAs in the ‘plant–pathogen interaction’ category (Table 2).

**Table 2.** The corresponding miRNAs in the plant–pathogen interaction KEGG pathway

	Fold change (log_2_ *spr2*/WT)	Predictive targets related to plant–pathogen interaction
Known miRNA		
miR1222a	–8.73233707	Solyc01g005440.2.1
miR2111a-5p	–10.16755622	Solyc06g083390.2.1
miR2593e	–7.90454432	Solyc04g051540.2.1
miR319b	1.29821124	Solyc03g115010.1.1
miR396b	–16.49819511	Solyc07g041640.2.1, Solyc10g083510.1.1, Solyc08g083230.1.1, Solyc08g075950.1.1, Solyc03g082430.1.1, Solyc08g005430.2.1, Solyc12g096070.1.1
miR476b	–8.31052191	Solyc11g064770.1.1, Solyc11g069620.1.1, Solyc11g071420.1.1
miR482c	–10.79691559	Solyc04g009110.1.1, Solyc04g009130.2.1, Solyc04g009290.1.1, Solyc04g026110.2.1, Solyc07g009180.1.1, Solyc08g075630.2.1, Solyc08g076000.2.1, Solyc09g098100.2.1
miR482d-3p	3.34641851	Solyc02g036270.2.1, Solyc04g009070.1.1, Solyc11g065780.1.1, Solyc12g016220.1.1
miR5081	–1.35827709	Solyc02g037540.1.1
miR5139	1.0785291	Solyc05g006620.2.1
miR5185l-5p	–10.43434683	Solyc01g106630.2.1, Solyc04g074000.2.1
miR5658	–10.71188213	Solyc06g068930.1.1
miR6020a-5p	–2.05955615	Solyc01g066020.1.1
miR6022	–1.05886924	Solyc01g005720.2.1, Solyc01g005730.2.1, Solyc01g005760.2.1, Solyc01g005780.1.1, Solyc01g005870.1.1, Solyc01g006550.2.1, Solyc01g008390.1.1, Solyc01g008410.1.1, Solyc01g009690.1.1, Solyc01g009700.1.1, Solyc03g082780.1.1, Solyc12g100020.1.1
miR6023	–1.2612632	Solyc01g005780.1.1, Solyc01g009690.1.1, Solyc03g082780.1.1, Solyc01g005710.2.1, Solyc01g014160.1.1, Solyc01g014930.1.1
miR6024	–3.44754718	Solyc02g070410.1.1, Solyc02g084890.1.1, Solyc03g006750.1.1, Solyc04g015220.2.1, Solyc05g005330.2.1, Solyc05g008070.2.1, Solyc11g006640.1.1, Solyc11g020100.1.1, Solyc11g069020.1.1, Solyc12g005970.1.1, Solyc12g006040.1.1, Solyc12g017800.1.1
miR6025a	–8.04204236	Solyc05g012890.1.1, Solyc05g012910.2.1, Solyc08g076050.2.1, Solyc08g076060.2.1
miR6027	1.71280122	Solyc04g007070.2.1, Solyc04g009090.1.1, Solyc04g009150.1.1
miR6426a	2.53010865	Solyc10g009210.2.1
miR6472	–1.97800646	Solyc05g044490.2.1
miR7819	–12.8364427	Solyc01g113620.1.1
miR900–3p	11.55914762	Solyc11g011880.1.1
Novel miRNA		
novel_mir_1138	9.8746433	Solyc04g056570.2.1
novel_mir_1167	8.21412481	Solyc07g047910.1.1
novel_mir_1248	8.25993153	Solyc07g049180.2.1
novel_mir_1297	7.58187845	Solyc05g007170.2.1, Solyc09g092000.2.1
novel_mir_1365	9.71937171	Solyc07g047910.1.1
novel_mir_17	–6.83048352	Solyc07g047910.1.1
novel_mir_607	1.56364113	Solyc05g007170.2.1, Solyc09g092000.2.1
novel_mir_1310	11.79131757	Solyc04g009110.1.1, Solyc04g009130.2.1, Solyc04g009290.1.1, Solyc04g026110.2.1, Solyc07g009180.1.1, Solyc08g075630.2.1, Solyc08g076000.2.1, Solyc09g098100.2.1
novel_mir_1379	8.0148016	Solyc03g113980.2.1
novel_mir_1432	11.49834063	Solyc10g047490.1.1, Solyc12g009510.1.1, Solyc12g009520.1.1, Solyc12g013680.1.1
novel_mir_181	5.25660878	Solyc01g087200.2.1, Solyc04g007320.1.1
novel_mir_288	–10.94453626	Solyc06g008270.2.1, Solyc12g006020.1.1, Solyc12g099870.1.1, Solyc12g100010.1.1, Solyc12g100030.1.1
novel_mir_313	–8.44057854	Solyc06g068870.2.1
novel_mir_318	–7.58263127	Solyc07g039570.2.1
novel_mir_366	–9.07446263	Solyc05g008070.2.1, Solyc07g049700.1.1, Solyc11g006530.1.1, Solyc11g006630.1.1, Solyc11g020100.1.1, Solyc12g017800.1.1
novel_mir_453	–10.12175366	Solyc07g053220.1.1
novel_mir_549	–6.97487343	Solyc09g092280.1.1
novel_mir_553	–9.42808795	Solyc05g021140.1.1, Solyc10g085460.1.1, Solyc10g086590.1.1
novel_mir_633	–13.11884318	Solyc03g078300.1.1, Solyc08g007630.1.1, Solyc11g043070.1.1, Solyc11g071430.1.1
novel_mir_657	–6.83048352	Solyc11g071420.1.1
novel_mir_683	–3.49249358	Solyc03g063650.1.1
novel_mir_71	2.91441758	Solyc01g005710.2.1, Solyc01g005730.2.1, Solyc01g005760.2.1, Solyc01g005780.1.1, Solyc01g005870.1.1, Solyc01g006550.2.1, Solyc01g009700.1.1, Solyc01g016370.1.1, Solyc03g082780.1.1, Solyc10g007210.1.1
novel_mir_906	8.50787391	Solyc07g018190.2.1, Solyc10g085120.1.1, Solyc12g044840.1.1

### Validation and characterization of selected miRNAs responsive to RKN infection in the WT and *spr2*

**Fig. 3.**
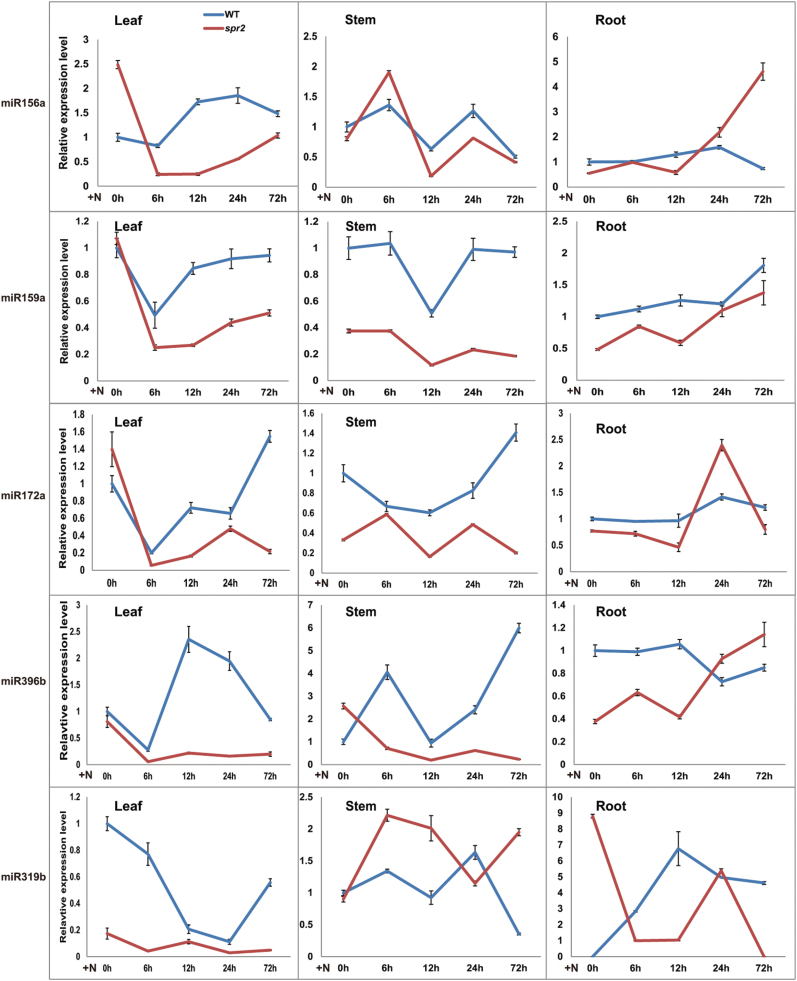
Time-course expression profiling of miRNAs (miR156a, miR159a, miR172a, miR396b, and miR319b) from leaves, stems, and roots at 0, 6, 12, 24, and 72h after RKN inoculation (N) in the WT and *spr2*. At least 10 plants were included in each pool, and three technical replicates were determined for each time point. Error bars indicate ±SE. The primary axis corresponds to the solid line, and the secondary axis corresponds to the dashed line. (This figure is available in colour at *JXB* online.)

miR156 (Kasschau *et al.*, 2003; Bazzini *et al.*, 2007; Lu *et al.*, 2007; Navarro *et al.*, 2008; Xin *et al.*, 2010; Zhang *et al.*, 2011), miR159 (Subramanian *et al.*, 2008; Xin *et al.*, 2010; Zhang *et al.*, 2011), miR172 (Subramanian *et al.*, 2008; Wang *et al.*, 2009; Zhang *et al.*, 2011), miR396 (Hewezi *et al.*, 2008, 2012; Navarro *et al.*, 2008; Wang *et al.*, 2009; Xin *et al.*, 2010; Zhang *et al.*, 2011), and miR319 (Subramanian *et al.*, 2008; Zhang *et al.*, 2011; Feng *et al.*, 2014; Shen *et al.*, 2014) have all been shown to respond to biotic stress in plants. Therefore, miRNA156a, miRNA159a, miRNA172a, miR319b, and miR396b were selected to validate the sRNA sequencing results and further characterize the spatio-temporal expression patterns in the WT and *spr2*. After RKN inoculation of the WT, dynamic transcription fluctuations in selected miRNAs appeared in the leaves, stems, and roots (Fig. 3), indicating that those miRNAs were responsive to RKN invasion. Using pre-RKN inoculation (0h) in the WT as the control, the expression levels of selected miRNAs in leaves, stems, and roots at 0, 6, 12, 24, and 72h after inoculation were analysed in the WT and *spr2*. Significant differences in miRNA expression between the WT and *spr2* were observed (Fig. 3), which was consistent with the sRNA sequencing results and suggested that JA signal deficiency affects the response of selected miRNAs under RKN infection.

### Expression profiling of selected miRNAs and their targets under RKN invasion

**Fig. 4.**
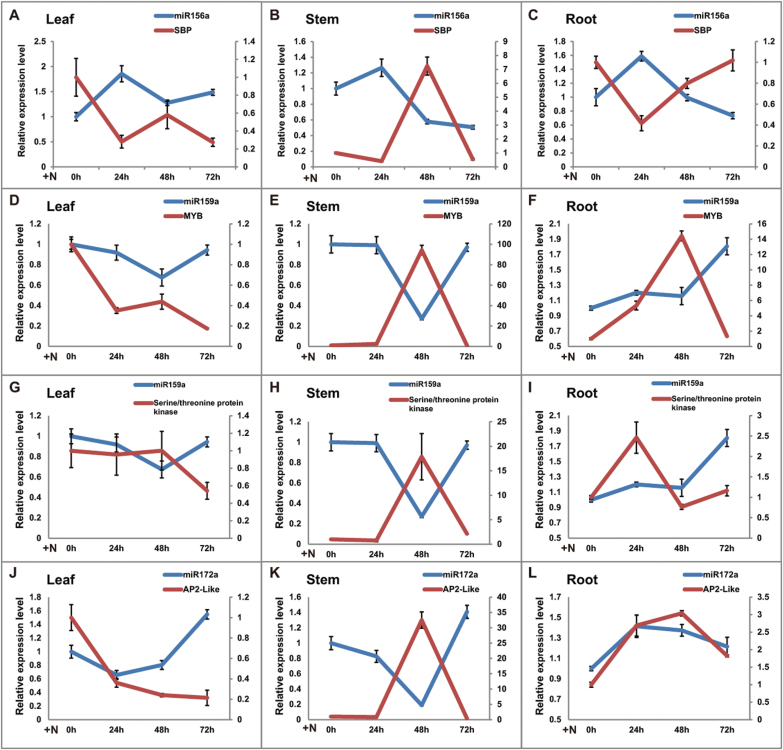
Time-course expression profiling of miRNAs and target genes from leaves, stems, and roots at 0, 24, 48, and 72h after RKN inoculation (N) in the WT. At least 10 plants were included in each pool, and three technical replicates were determined for each time point. Error bars indicate ±SE. The primary axis corresponds to the solid line, and the secondary axis corresponds to the dashed line. (This figure is available in colour at *JXB* online.)

To investigate the potential role of selected miRNAs in response to RKN, the spatio-temporal expression profiling of predicted targets of miR156a, miR159a, and miR172a was also examined in the WT after RKN infection. The squamosa promoter-binding protein gene (Solyc10g078700.1.1, *SPL*) is the target of miR156 (Lu *et al.*, 2005, 2008; Bazzini *et al.*, 2007). An opposite expression pattern between miR156a and *SPL* was observed in leaf (0–24h), stem (0–48h), and root (0–72h) (Fig. 4A–C). The *MYB* transcription factor is reportedly targeted by miR159 (Achard *et al.*, 2004; Sunkar *et al.*, 2004; Reyes and Chua, 2007; Liu *et al.*, 2008), and a serine/threonine protein kinase (Solyc06g008320.2.1) was also predicted to be targeted by miR159a in the present analysis. Compared with miR159a, the MYB transcription factor (Solyc01g009070.2.1) showed a reverse expression pattern after 48h in leaf and root (Fig. 4D, F), and from 24h to 72h in stem (Fig. 4E). A negative correlation was found between the expression of the serine/threonine protein kinase gene and miR159a from 24h to 72h in stem (Fig. 4H), and no negative correlation was observed in leaf and root (Fig. 4G, I). The AP2-like ethylene-responsive transcription factor is the target of miR172 (Aukerman and Sakai, 2003; Chen *et al.*, 2004; Schwab *et al.*, 2005), and miR172a and AP2-like transcription factor gene (Solyc04g049800.2.1) expression showed a reverse pattern from 0h to 72h in leaf and from 24h to 72h in stem (Fig. 4J, K), and analogous patterns occurred in root (Fig. 4L). The fact that these miRNA–target pairs exhibited inverse expression at particular times and places suggests that miRNAs potentially play roles in self-adaption to RKN invasion through the regulation of their target genes.

### *miR319*- and *LA* ^*m*^-overexpressing transgenic tomatoes

**Fig. 5.**
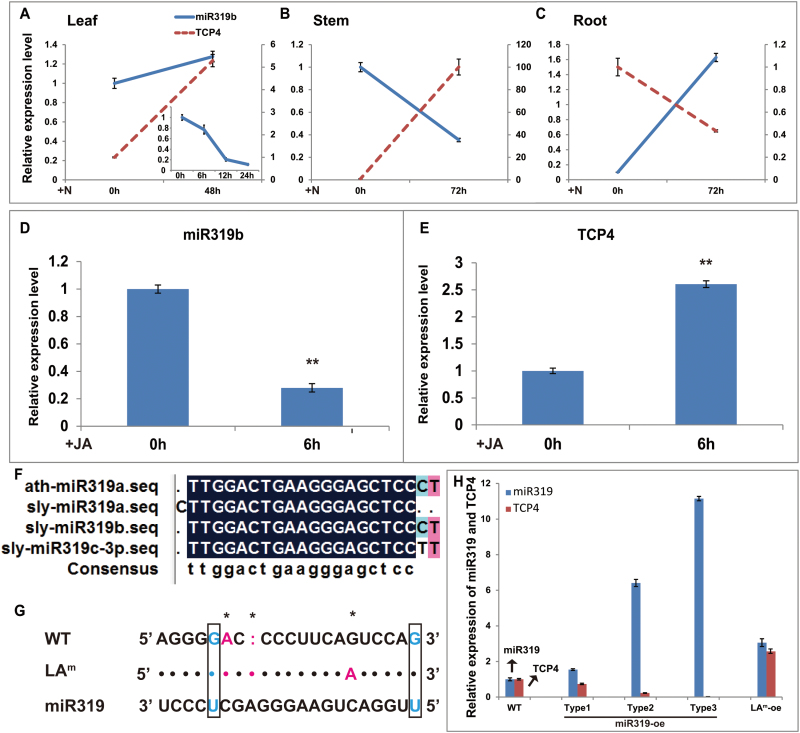
(A–C) Changes in *miR319b* and *TCP4* expression in leaves, stems, and roots after RKN inoculation (N) in the WT. The primary axis corresponds to the solid line, and the secondary axis corresponds to the dashed line. The mini line chart shows the dynamic changes of miR319b from 0h to 24h after RKN inoculation. (D, E) Changes in *miR319b* and *TCP4* expression in leaves after JA treatment in the WT. The asterisk represents a significant difference as determined by Student’s *t*-test (*P*≤0.01). (F) The sequence similarity alignment between ath-miR319a in *Arabidopsis* and sly-miR319a, b, c in tomato. (G) Sequence of the miR319 recognition site in the *LA* (*TCP4*) mRNA of the WT and LA^m^. The box and asterisk represent G–U wobbles and mismatches, respectively. (H) Relative expression levels of *miR319* and *LA* in the leaves of the WT and miR319-oe, LA^m^-oe plants. At least 10 plants were included in each pool, and three technical replicates were determined for each time point. Error bars indicate ±SE. (This figure is available in colour at *JXB* online.)

In the present study, miR319b was responsive to RKN invasion (Fig. 3) and was predicted to be involved in plant–pathogen interaction (Table 2). *TCP4*, the target of miR319 (Palatnik *et al.*, 2003; Koyama *et al.*, 2007; Ori *et al.*, 2007; Efroni *et al.*, 2008), showed a significant up-regulation in leaf, though no obvious expression changes in miR319b were observed from 0h to 48h; however, miR319b expression dramatically declined from 0h to 24h (Fig. 5A). The reverse expression trends between *TCP4* and miR319b were observed in stem and root (Fig. 5B, C). Although the fold change of miR319b expression in sRNA sequencing was not the highest, the plausible miR319b-targeted inhibition of TCP4 occurred in both shoots and roots. For JA treatment, the expression of miR319b declined, and TCP4 increased (Fig. 5D, E). Based on all the above, an attempt was made to identify the function of miR319 during RKN invasion. *LA* (*LANCEOLATE*) encodes a TCP-family transcription factor (TCP4) that contains a miR319-binding site in tomato, which could be cleaved and down-regulated by ath-miR319a from *Arabidopsis* (Ori *et al.*, 2007). A sequence comparison of ath-miR319a (*Arabidopsis*) and sly-miR319s (tomato) showed that ath-miR319a and sly-miR319b are identical (Fig. 5F), indicating that ath-miR319a could function in place of sly-miR319b in tomato. To study the potential function of miR319b in response to RKN stress in tomato, the transactivation system was used to express ath-miR319a and *TCP4* (*LA*) under the regulation of the FILAMENTOUS FLOWER (FIL) promoter in the transgenic M82 tomato line. The FIL promoter is active primarily in tomato young primordia and later in initiating leaflets (Lifschitz *et al.*, 2006). The driver line expressed *LhG4* under the regulation of the *FIL* promoter (*FILpro:LhG4*) and the responder line expressed miR319 or *TCP4* under the control of an operator (OP) array. *FILpro:LhG4* (*pFIL*) plants were crossed with the *op:gene* plants (*op-miR319* or *opLA* ^*m*^) to obtain *miR319* or *LA* ^*m*^ overexpression plants (*FIL>>miR319*, miR319-oe; *FIL>>LA* ^*m*^, LA^m^-oe). The *LA* ^*m*^ contains a mutation in the *LA* miRNA-binding site that is complementary to miR319 and is thus a miR319-resistant version of *LA* (Fig. 5G). Finally, three *miR319* overexpression types were obtained, and the expression levels of miR319 and *TCP4* in hybrid F_1_ were analysed by qRT–PCR (Fig. 5H). Based on the significance of the expression level, type 3 was chosen for the following tests.

### *miR319* overexpression reduces resistance to RKN

**Fig. 6.**
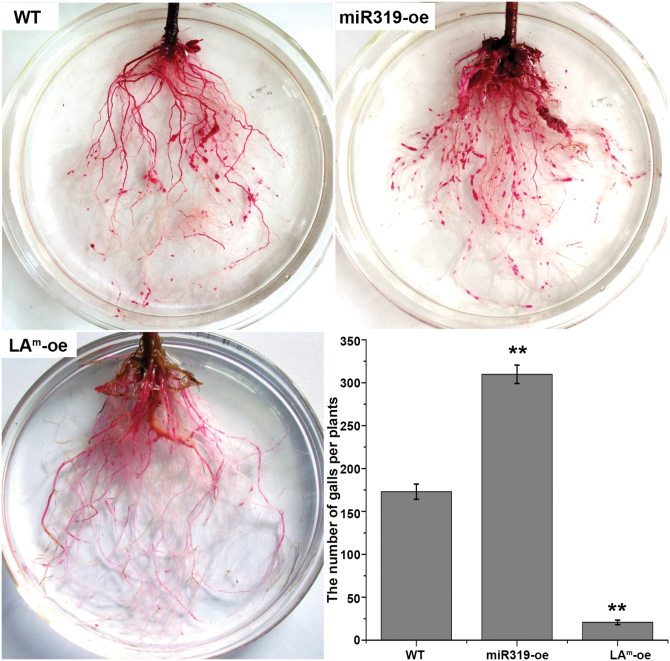
Representative root systems at 35 d after RKN infection, as well as the number of galls in WT, miR319-oe, and LA^m^-oe tomatoes. Three independent experiments were conducted, and 10 plants for each type were determined. Error bars indicate ±SD. (This figure is available in colour at *JXB* online.)

Previous studies reported that the overexpression of miR319 plays a positive role in various abiotic stress responses. For example, miR319 overexpression leads to enhanced cold tolerance in rice (Yang *et al.*, 2013). Zhou *et al.* (2013) revealed that miR319 overexpression enhances salt and drought tolerance in transgenic creeping bentgrass. In this study, WT and transgenic tomatoes were examined for their resistance to RKN infection. Tomato roots were inoculated with RKN and then stained with acid fuchsin at 35 d after inoculation, producing obvious red staining of the galls, and the number of galls per plant was then evaluated. The results indicated that the number of galls on miR319-oe roots was much higher than on WT roots; however, the LA^m^-oe plants had few galls (Fig. 6). This finding indicated that miR319 and TCP4 had opposite effects on resistance to RKN infection.

### *miR319* overexpression reduces endogenous JA levels

**Fig. 7.**
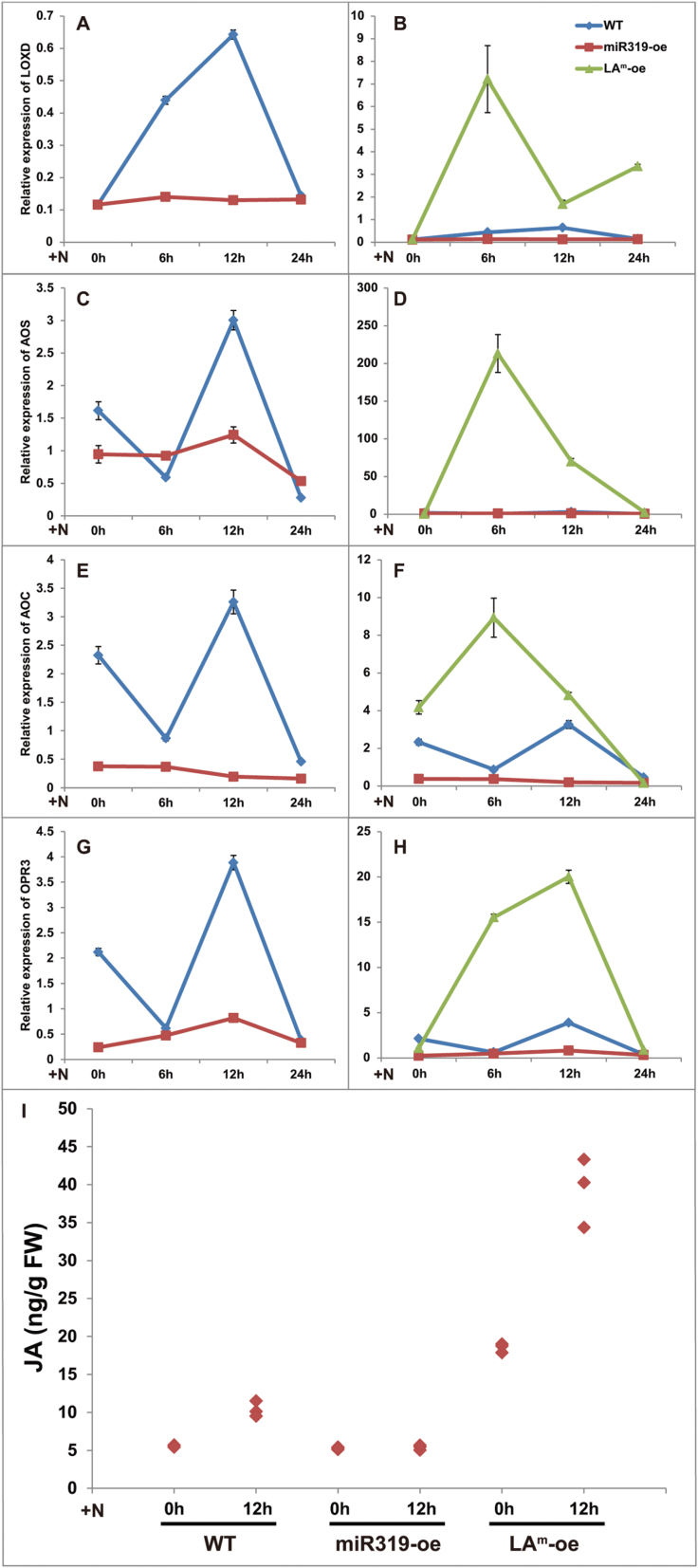
(A–H) Relative expression levels of JA biosynthetic genes in WT and transgenic tomatoes (miR319-oe and LA^m^-oe) at 0, 6, 12, and 24h after inoculation. The expression patterns of *LOXD* (A, B), *AOS1* (C, D), *AOC1* (E, F), and *OPR3* (G, H) are shown. At least 10 plants were included in each pool, and three technical replicates were determined for each time point. Error bars indicate ±SE. (I) Endogenous JA levels in the WT, miR319-oe, and LA^m^-oe lines. The JA concentration in leaves was measured in triplicate at 0h and 12h after inoculation. At least 10 plants were included in each pool, and three biological replicates were performed. (This figure is available in colour at *JXB* online.)

JA is a plant signalling compound that induces resistance to pathogens (Cooper *et al.*, 2005). Schommer *et al.* (2008) showed that JA biosynthesis is associated with the miR319/TCP regulatory module in *Arabidopsis*. Moreover, the JA biosynthesis and signalling pathways play key roles in the RKN resistance of the rice root system (Cooper *et al.*, 2005; Fujimoto *et al.*, 2011; Nahar *et al.*, 2011). In the present study, the expression levels of the pivotal genes involved in JA biosynthesis, including *LOXD*, *AOS1*, *AOC1*, and *OPR3*, were monitored in WT and transgenic tomatoes at 0, 6, 12, and 24h after RKN inoculation. *LOXD* expression in the WT line peaked at 12h after inoculation, but no obvious change was observed in miR319-oe (Fig. 7A). In the LA^m^-oe plants, *LOXD* expression peaked at 6h after inoculation (Fig. 7B). Moreover, similar expression patterns for *AOS1*, *AOC1*, and *OPR3* were found in the WT; they first decreased and then peaked at 12h after inoculation. However, no obvious changes were observed in the miR319-oe plants (Fig. 7C, E, G). In LA^m^-oe, similar to *LOXD*, the expression levels of *AOS1* and *AOC1* peaked at 6h after inoculation (Fig. 7D, F), and the expression level of *OPR3* peaked at 12h after inoculation (Fig. 7H). These results showed that the expression levels of JA biosynthetic genes in miR319-oe were low, and no obvious change was observed after RKN inoculation. However, those genes accumulated earlier and to a greater extent in LA^m^-oe plants, suggesting that the miR319/TCP4 module plays crucial roles in modulating JA biosynthesis induced by RKN invasion. In addition, the co-expression of *LOXD*, *AOS1*, *AOC1*, and *OPR3* in WT and LA^m^-oe suggested that those genes belonging to the LOX pathway are probably under the control of the same regulatory mechanism.

The levels of endogenous JA in WT and transgenic tomatoes were further detected at 0h and 12h after inoculation. The results showed that RNK invasion increased the endogenous JA levels in the WT but not in miR319-oe plants. *LA* ^*m*^ overexpression led to a remarkable increase in basal and induced JA levels (Fig. 7I).

### Changes in expression of miR396 in roots under RKN stress

**Fig. 8.**
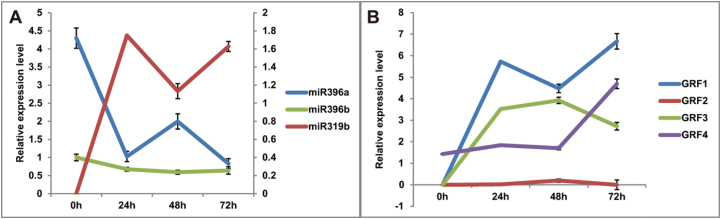
The relative expression levels of miR396a, miR396b, miR319b (A), and *GRF* (B) genes in roots at different time points after RKN inoculation in the WT. The primary axis corresponds to the lines with diamonds and triangles and the secondary axis corresponds to the line with squares. At least 10 plants were included in each pool, and three technical replicates were determined for each time point. Error bars indicate ±SE. (This figure is available in colour at *JXB* online.)

Previous studies reported that miR396/GRF functioned in nematode resistance in the roots of *Arabidopsis* (Hewezi *et al.*, 2012). The sequencing data showed that both miR396a and miR396b were differentially expressed between the two libraries (Supplementary Table S2 at *JXB* online). miR396a expression levels declined in root; however, no obvious expression differences were observed for miR396b (Fig. 8A). The miR396 targets *GRF1*, *GRF3*, and *GRF4*, but not *GRF2*, were transcriptionally up-regulated (Fig. 8B), suggesting that the miR396/GRF module is responsive to RKN invasion in tomato.

## Discussion

### miR319 negatively regulates RKN resistance by affecting the JA level in plants

miR319 is one of the most highly conserved miRNAs in plants and was also one of the first to be identified (Weigel *et al.* 2000; Palatnik *et al.* 2003; Schommer *et al.* 2012). Previous studies revealed that the miR319 family is involved in biotic stress response (Zhang *et al.*, 2011; Feng *et al.*, 2014; Shen *et al.*, 2014). In the present work, miR319 was identified between two libraries, WT+N and *spr2*+N (Supplemetnary Table S2 at *JXB* online), and the expression analysis indicated that the response of miR319/TCP4 to RKN invasion occurred in both shoots and roots (Fig. 5A–C). miR319 has been reported to target *TCP* genes, which encode plant-specific transcription factors (Palatnik *et al.*, 2003, 2007; Schwab *et al.*, 2005; Koyama *et al.*, 2007, 2010; Ori *et al.*, 2007; Schommer *et al.*, 2008, 2012; Nag *et al.*, 2009) that are involved in JA biosynthesis and senescence (Schommer *et al.*, 2008). Microarray experiments comparing the shoot apical meristem transcriptomes of WT and miR319-oe plants have revealed a clear decrease in the levels of all miR319-targeted *TCP* genes (Palatnik *et al.*, 2003; Efroni *et al.*, 2008; Schommer *et al.*, 2008). Consistent with these studies, miR319 overexpression led to a decrease in the transcriptional level of *TCP4* (Fig. 5H). Schommer *et al.* (2008) and Hao *et al.* (2012) demonstrated that *TCP* positively regulates the JA level in plants. Similarly, it was also observed here that *LA* ^*m*^ overexpression resulted in an increase in the basal and RKN-induced levels of JA-synthetic gene expression and endogenous JA; however, these levels were decreased in the miR319-oe plants (Fig. 7). Previous studies revealed that the JA pathway is a crucial player in maintaining and defending RKN in plants (Cooper *et al.*, 2005; Fujimoto *et al.*, 2011; Nahar *et al.*, 2011; Fan *et al.*, 2014). In the present study, RKN resistance increased in LA^m^-oe but decreased in the miR319-oe plants (Fig. 6). Taken together, these results suggested that overexpressing miR319 inhibited *TCP4*, which in turn regulated JA biosynthetic genes (Fig. 7A–H), resulting in a lower endogenous JA level (Fig. 7I). Consequently, the resistance to RKN infection was affected in miR319-oe tomatoes (Fig. 6).

**Fig. 9.**
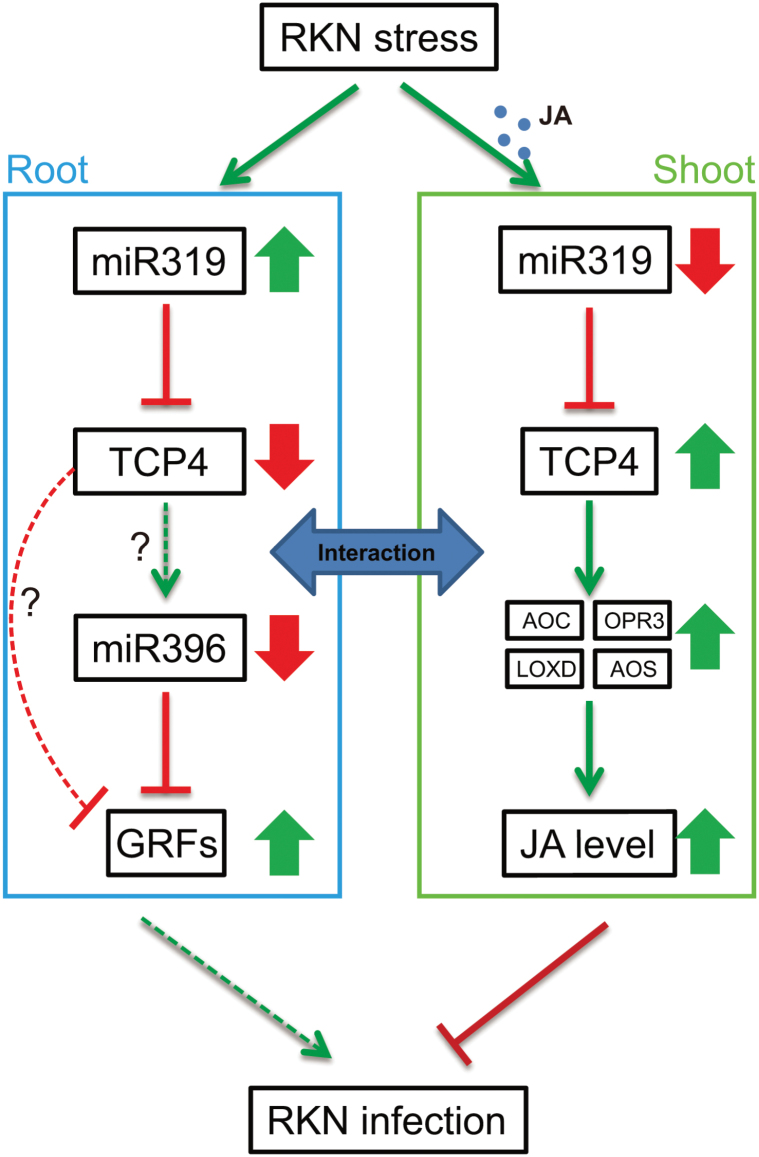
Model of miR319/TCP-mediated regulation of RKN resistance in the WT background. Small filled circles indicate JA molecules. Solid lines represent regulatory links observed in tomato, and dashed lines represent regulatory links observed in *Arabidopsis*. Arrows indicate positive regulation, and blunt-ended bars indicate inhibition. A line does not necessarily represent unique or direct regulation. A question mark refers to unverified regulation under RKN stress. Broad arrows represent the up- or down-regulation of genes and hormone levels. (This figure is available in colour at *JXB* online.)

In the WT background, JA treatment led to the decrease of expression of miR319b and increase of TCP4 (Fig. 5D, E) in leaves, which is consistent with the observations in RKN stress (Fig. 5A). Combined with the results in *spr2* (Fig. 3), these findings implied that JA acts as the regulator of miR319b in early RKN response (Fig. 9). miR319b was responsive to RKN invasion and was down-regulated in shoot, with the expression of *TCP4* subsequently increasing (Fig. 5A, B), such that JA synthesis in shoots may become activated (Fig. 7A, C, E, G, I), leading to JA-mediated systemic resistance (Fig. 9). Combined with the results in miR319-oe and LA^m^-oe, this suggested that JA mediated miR319 serving as a systemic defensive responder and modulator that functioned at least partially via TCP4 under RKN stress (Fig. 9).

### miR396 probably participates in RKN resistance in tomato roots

miR396 is a negative regulator of mitotic cell division that acts through the down-regulation of *GRF* genes in shoot meristems, leaves, and roots (Liu *et al.*, 2008; Rodriguez *et al.*, 2010; Wang *et al.*, 2011; Hewezi *et al.*, 2012). The miR396/GRF module was reported to be involved in cyst nematode resistance in *Arabidopsis* (Hewezi *et al.*, 2008, 2012). The accumulated evidence demonstrates that miR319 and miR396 are the miRNAs with the most molecular connections to phytohormone signalling pathways (Curaba *et al*., 2014). The overexpression of an miRNA-resistant form of *AtTCP4*, a target of miR319, results in increased miR396 accumulation and a corresponding decrease in *AtGRF* gene expression, and *AtTCP4* also regulates GRF activity independently of miR396 (Rodriguez *et al.*, 2010; Fig. 9). Recently, Schommer *et al.* (2014) reported that miR319-regulated TCP4 positively regulates miR396, thereby repressing GRFs and cell proliferation in *Arabidopsis*. In the present study, the expression patterns between miR319b and miR396a were diametrically opposite (Fig.5A), hence a similar regulation between miR396a and miR319b/TCP4 appeared to occur in tomato roots under RKN stress (Figs 8A, 9). Previous studies demonstrated that miR396 was significantly down-regulated in response to cyst nematodes, which is consistent with the present results (Fig. 8A; Hewezi *et al.*, 2008), and miR396 overexpression reduced the syncytium size and arrested cyst nematode development by repressing GRFs in *Arabidopsis* roots (Fig. 9; Hewezi *et al.*, 2012).

Based on the above data, a model is proposed whereby the miR319/TCP module influences RKN resistance via two pathways in tomato (Fig. 9): TCP-regulated JA biosynthesis in shoots and cell proliferation regulated by the miR396/GRF module in roots.

## Supplementary data

Supplementary data are available at *JXB* online.

Figure S1. *Mi-1* gene test of plant materials used in this study.

Table S1. Primers used for stem–loop RT–-PCR and qRT–PCR.

Table S2. Differentially expressed known miRNAs between the WT and *spr2* after RKN inoculation.

Table S3. Differentially expressed novel miRNAs between the WT and *spr2* after RKN inoculation.

Table S4. Predicted targets of differentially expressed known miRNAs.

Table S5. Predicted targets of differentially expressed novel miRNAs.

Table S6. KEGG enrichment of known and novel miRNA targets.

Supplementary Data
