# Supplementary material for: Identification of jasmonic acid-associated microRNAs and characterization of the regulatory roles of the miR319/TCP4 module under root-knot nematode stress in tomato
Source: J Exp Bot. 2015 May 22;66(15):4653–67. doi: 10.1093/jxb/erv238 (PMC4507771; doi:10.1093/jxb/erv238)
Supplement: Supplementary Data [file supp_66_15_4653__index.html]

Identification of jasmonic acid-associated microRNAs and characterization of the regulatory roles of the miR319/TCP4 module under root-knot nematode stress in tomato — Identification of jasmonic acid-associated microRNAs and characterization of the regulatory roles of the miR319/TCP4 module under root-knot nematode stress in tomato — Supplementary Data 

# Identification of jasmonic acid-associated microRNAs and characterization of the regulatory roles of the miR319/TCP4 module under root-knot nematode stress in tomato

## Supplementary Data

Data files

**Files in this Data Supplement:**

- Supplementary Data - Supplementary Data
- Supplementary Data - Supplementary Data
- Supplementary Data - Supplementary Data
