# Supplementary material for: Identification of jasmonic acid-associated microRNAs and characterization of the regulatory roles of the miR319/TCP4 module under root-knot nematode stress in tomato
Source: J Exp Bot. 2015 May 22;66(15):4653–67. doi: 10.1093/jxb/erv238 (PMC4507771; doi:10.1093/jxb/erv238)
Supplement: Supplementary Data [file supp_erv238_jexbot148080_file002.pdf]

Table S1 The primers of stem-loop RT-PCR and qRT-PCR

miRNA	Primers
miR156a	RT: GTTGGCTCTGGTGCAGGGTCCGAGGTATTCGCACCAGAGCCAACGTGCTC<br>F: CGGCGGTTGACAGAAGATAG & R: GTGCAGGGTCCGAGGTATTC
miR159a	RT: GTTGGCTCTGGTGCAGGGTCCGAGGTATTCGCACCAGAGCCAACTAGAGC<br>F: CGGCGGTTTGGATTGAAGGG & R: GTGCAGGGTCCGAGGTATTC
miR172a	RT: GTTGGCTCTGGTGCAGGGTCCGAGGTATTCGCACCAGAGCCAACATGCAG<br>F: CGGCGAGAATCTTGATGATG & R: GTGCAGGGTCCGAGGTATTC
miR396a	RT: GTCGTATCCAGTGCAGGGTCCGAGGTATTCGCACTGGATACGACTTCCCAC<br>F: CGCCACAGTTCAATAAAGCT & R: TCGTATCCAGTGCAGGGTC
miR396b	RT: GTCGTATCCAGTGCAGGGTCCGAGGTATTCGCACTGGATACGAAAGTTCAA<br>F: GTCAGCATTCCACAGCTTTC & R: TCGTATCCAGTGCAGGGTC
miR319b	RT: GTCGTATCCAGTGCAGGGTCCGAGGTATTCGCACTGGATACGAGGGAGCTC<br>F: GCATTGGATTGGACTGAAGG & R: TCGTATCCAGTGCAGGGTC
U6	F: TCTAACAGTGTAGTTTGTCCCTTCG<br>R: TTGTGCGTGTCATCCTTGC

miRNAs	Targets	Primers
	β-actin	F: GGAATGGGACAGAAGGAT<br>R: CAGTCAGGAGAACAGGGT
miR396	Solyc07g041640.2.1 (GRF1)	F: CAGATGGAAAGAAATGGA<br>R: TGTTGATGATGATGTGTT
miR396	Solyc02g092070.2.1 (GRF2)	F: GGTCACTCTACTATGCTCAG<br>R: AGAATCAAACAATGGGAAAGG
miR396	Solyc10g083510.1.1 (GRF3)	F: AACCAACAACATCAACAACA<br>R: CCACCACTTCCAATCCTT
miR396	Solyc08g005430.2.1 (GRF4)	F: GGTTCTTCAGTCATCCTA<br>R: GTCCTTCTACATCTTCCT
miR156a	Solyc10g078700.1.1 (SBP)	F: TGTTGTTGCTGGTCTTGA<br>R: CTTCCTACGACGCTCATT
miR159a	Solyc01g009070.2.1 (MYB)	F: CTCATTCGGCTCTTCCTT<br>R: ATTACCAGATGTCTCGTTCC
miR159a	Solyc06g008320.2.1 (Serine/threonine protein kinase)	F: GACCTACAGACTCACAAGCATA<br>R: CATAAGCACATAGCCAAGAGATTC
miR172a	Solyc04g049800.2.1 (AP2-Like ERF)	F: ATATGCTCACCAGACAACTT<br>R: AATGTAACGCCACGATACT
miR319b	Solyc03g115010.1.1 (TCP4)	F: CTGATGCTATTGCTGATA<br>R: GCTATTGGCTCTTGATAA

JA related genes & Mi	Primers
LOXD	F: CATTGCCATTGAACTTAG<br>R: GAACTCCAGCATCATTAG
AOS1	F: CATCGTCTTATCGTGTTA<br>R: GAAGTAATCAAGTCTGTCT
AOC1	F: ACTTACTACCACCTCTAC<br>R: AGTGTTAGTTGAATCTGTT
OPR3	F: CACGGTTACTTGATTGAT<br>R: CTATTGCTGAGACTACTG
Mi-1	F: GCAATTCTAGATCTAGCTATTTGTTGTTC<br>R: CCTGCTCGTTTACCATTACTTTTCCAACC
