# Supplementary figures and images for: Identification of jasmonic acid-associated microRNAs and characterization of the regulatory roles of the miR319/TCP4 module under root-knot nematode stress in tomato
Source: J Exp Bot. 2015 May 22;66(15):4653–67. doi: 10.1093/jxb/erv238 (PMC4507771; doi:10.1093/jxb/erv238)

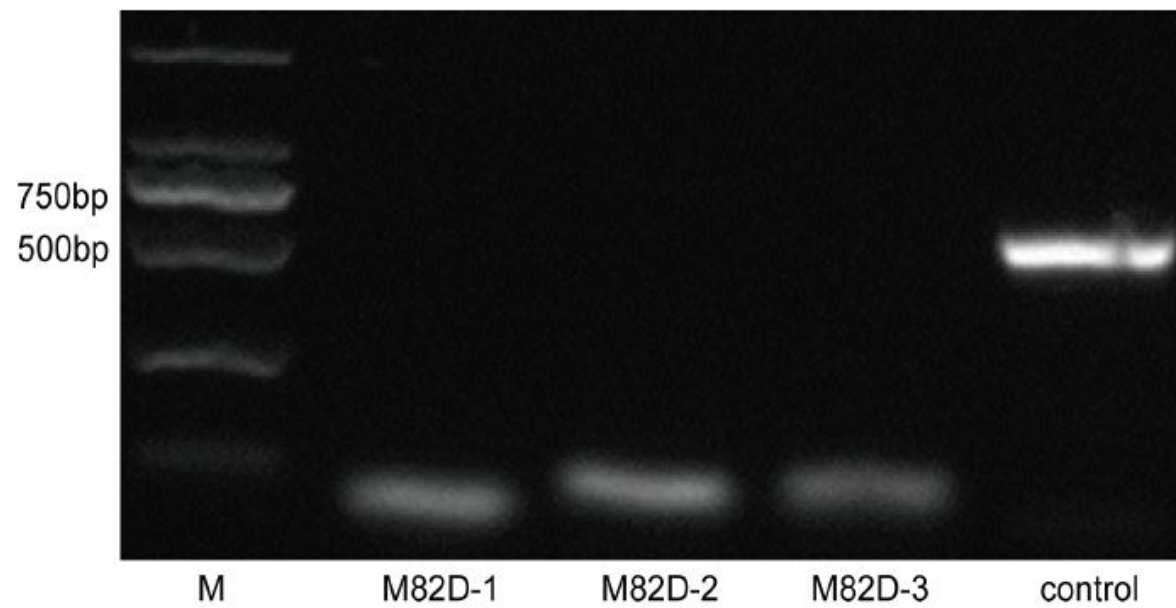

Supplement: Supplementary Data [file supp_erv238_jexbot148080_file001.pdf]
